# Supplementary material for: Facile Fabrication of Microfluidic Chips for 3D Hydrodynamic Focusing and Wet Spinning of Polymeric Fibers
Source: Polymers (Basel). 2020 Mar 10;12(3):633. doi: 10.3390/polym12030633 (PMC7182802; doi:10.3390/polym12030633)

Supplementary Materials

Facile fabrication of microfluidic chips for 3D hydrodynamic focusing and wet spinning of polymeric fibers

Akin Gursoy[1], Kamran Iranshahi[1,†], Kongchang Wei[1], Alexis Tello[1], Efe Armagan[1], Luciano F. Boesel[1], Fabien Sorin[2], René M. Rossi[1], Thijs Defraeye[1], Claudio Toncelli[1,*]

1 Empa, Swiss Federal Laboratories for Materials Science and Technology, Laboratory for Biomimetic Membranes and Textiles, Lerchenfeldstrasse 5, CH-9014 St.Gallen; akin.gursoy@empa.ch (A.G.); kamran.iranshahi@empa.ch (K.I.); kongchang.wei@empa.ch (K.W.); talexis@student.ethz.ch (A.T.); efe.armagan@empa.ch (E.A.); Luciano.Boesel@empa.ch (L.F.B.); rene.rossi@empa.ch (R.M.R.); Thijs.Defraeye@empa.ch (T.D.); claudio.toncelli@empa.ch (C.T.)

2 Laboratory of Photonic Materials and Fibre Devices (FIMAP), Institute of Materials, Ecole Polytechnique Fédérale de Lausanne (EPFL), Lausanne 1015, Switzerland; fabien.sorin@epfl.ch (F.S.)

* Correspondence: claudio.toncelli@empa.ch (C.T.); Tel.: +41 58 765 7215

† Equal Contribution

S1. Multichannel PDMS spinneret

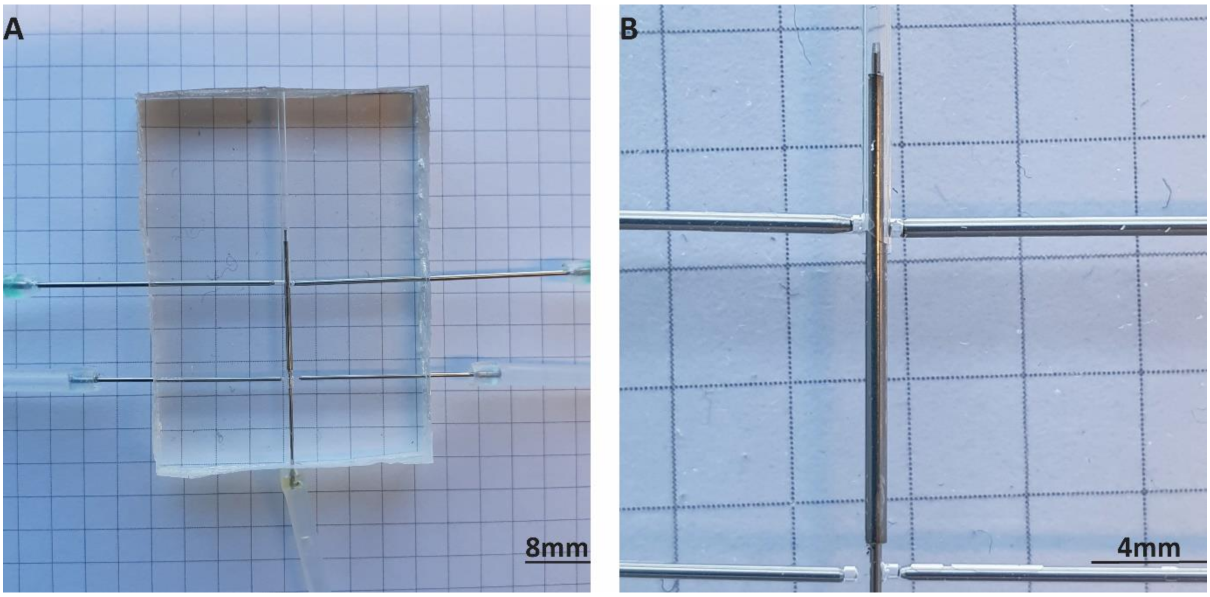

Figure S1: Multichannel reactor for double-coaxial flow focusing. **A**) Microfluidic setup. **B**) Nozzle assembly.

S2. Backflow simulation

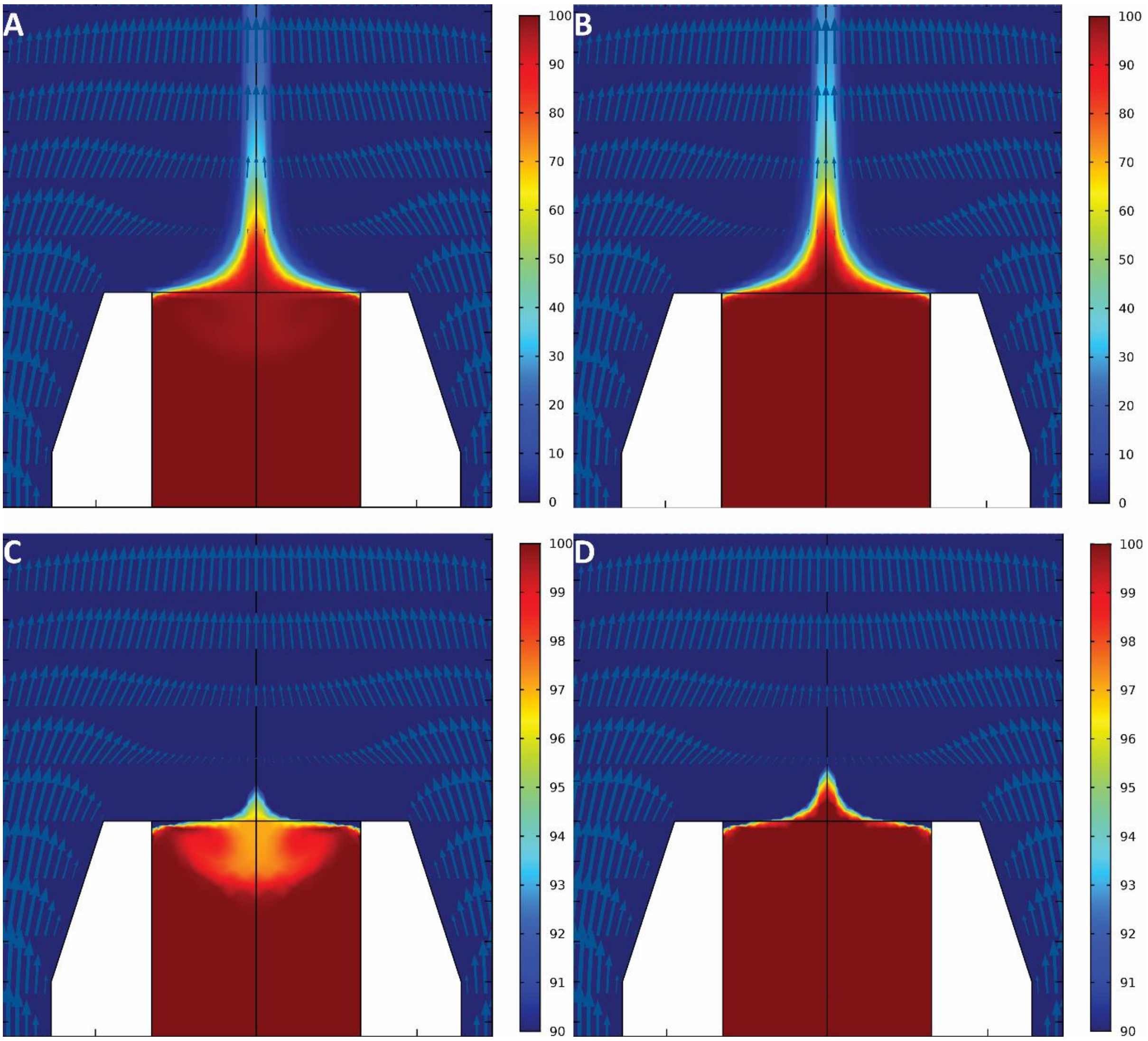

Figure S2: Backflow simulation (scale bar indicating solute concentration) under different flow conditions. Sheath and core flow rate was set as (**A**) 297 μL/min and 3 μL/min, respectively; **B**) 296 μL/min and 4 μL/min, respectively. (**C-D**) Scaled versions (concentration range from 90 to 100%) of figures in panel A and B, indicating the beginning of backflow at r = 100.

S3. mini-wet-spinner

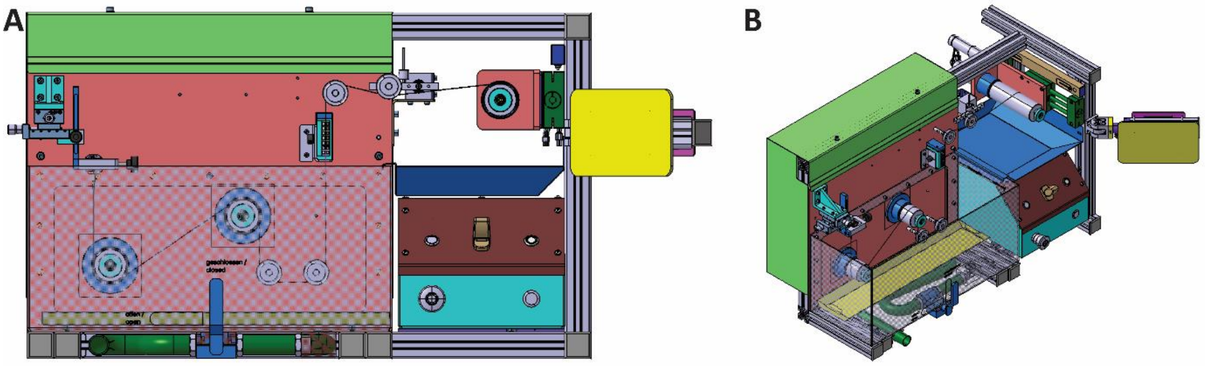

Figure S3: In house designed mini-wet-spinner.

S4. Backflow simulation

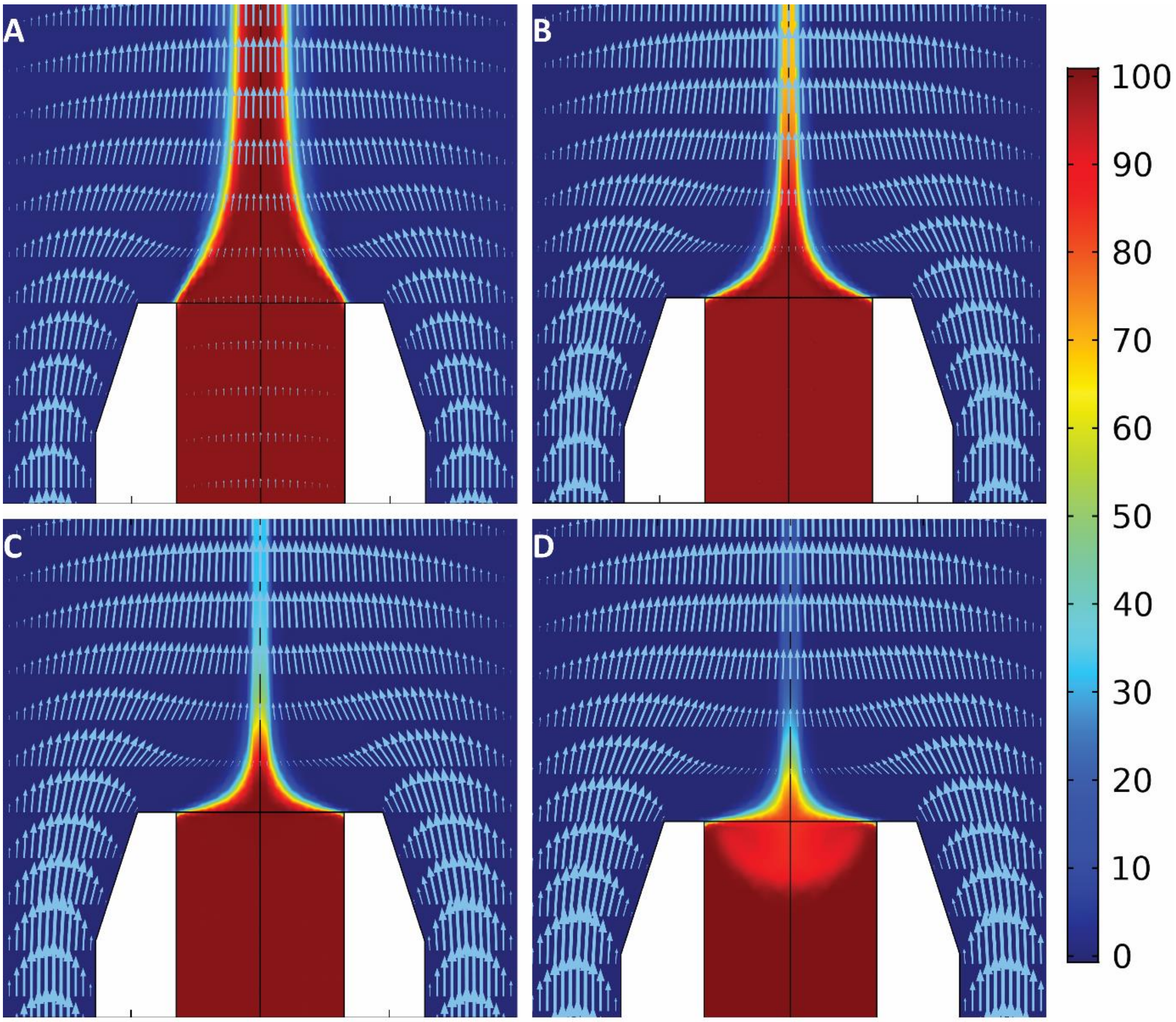

Figure S4: Concentration gradient simulation of hydrodynamic focussing indicating increased homogeneity of core flow with increased sheath/core flow ratio. (**A**) r = 2. (**B**) r = 20. (**C**) r = 50. (**D**) r = 100.

Computational model

The schematic illustration of the computational model together with the applied boundary conditions is depicted in S5. The computational domain dimensions are indicated in this figure. The outlet is positioned at a distance of 15 mm from the inlets that based on the laminar nature of the flow allows full flow development after mixing. The computational grid was constructed based on a grid-sensitivity analysis. The high grid resolution is applied in the proximity of the regions with expected large flow gradients. In this regard, grid refinement has been performed toward the core needle as well as the measuring section to capture these gradients accurately. The grid of the base case consists of 31373 hybrid (tetrahedral and quadrilateral) cells for example.

At the inlets of the domain, a uniform, normal velocity is imposed. A zero static pressure together with suppressed backflow is imposed at the outlet of the domain. Centreline is

considered as axial symmetry and the rest of the domain is considered as a no-slip wall. The steady Navier-Stokes and continuity equations are solved in combination with convection-diffusion equations for transport of diluted species. The microfluidics module of COMSOL was used, using a segregated solver, relying on the PARDISO (PARallel DIrect sparse SOlver Interface) solver scheme. Pressure and velocity interpolation are standard and the first-order discretization is used for both of them. Validation for higher total flow rate (1000 ul/min) is provided in S.6.

S5. Schematic of the computational model and the applied boundary conditions

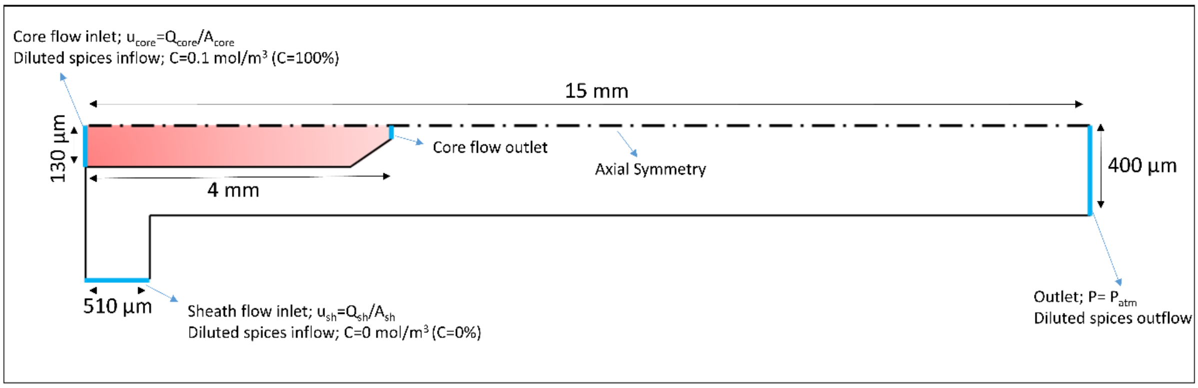

S6. Experimental and simulation results for total flow rate of 1000 ul/min

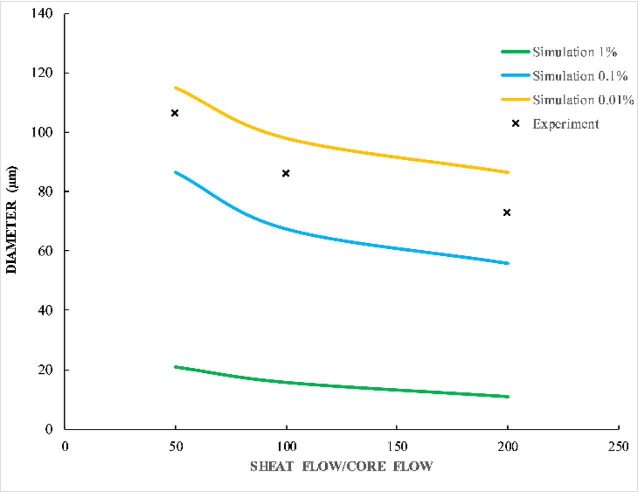

Supplement: Supplementary file 1 [file polymers-12-00633-s001.pdf]
